# Effectiveness of a three-component intervention supporting unemployed individuals with mental health issues in their job search and mental health recovery (3for1): study protocol of a non-randomized controlled study

**DOI:** 10.1186/s12889-024-20323-0

**Published:** 2024-11-14

**Authors:** Svenja Schlachter, Sophia Helen Adam, Maximilian Baxendale, Melanie Gantner, Maria Gralla, Florian Junne, Peter Martus, Miriam Mehler, Daniel Nischk, Marina Pumptow, Rebecca Erschens, Harald Gündel, Nicolas Rüsch, Jörn von Wietersheim

**Affiliations:** 1 Department of Psychiatry and Psychotherapy II, Section Public Mental Health, Ulm University and BKH Günzburg, Parkstraße 11, Ulm, 89073 Germany; 2 Department of Psychosomatic Medicine and Psychotherapy, Ulm University Medical Center, Albert-Einstein-Allee 23, Ulm, 89081 Germany; 3 Department of Psychosomatic Medicine and Psychotherapy, University Hospital Tübingen, Osianderstraße 5, Tübingen, 72076 Germany; 4 Department of Psychosomatic Medicine and Psychotherapy, Otto Von Guericke University Magdeburg, University Hospital Magdeburg, Leipziger Straße 44, Magdeburg, 39120 Germany; 5 Institute for Clinical Epidemiology and Applied Biometry, University Hospital Tübingen and Medical Faculty, University of Tübingen, Silcherstr. 5, Tübingen, 72076 Germany; 6 Department of Social Psychiatry, Reichenau Centre of Psychiatry, Feursteinstraße 55, Reichenau, 78479 Germany

**Keywords:** Mental health, Psychological distress, Vocational rehabilitation, Labor market integration, Unemployment, Individualized Placement and Support, Supported employment, Peer support, Peer navigator, Intervention

## Abstract

**Background:**

There is a vicious cycle between unemployment and mental health issues. Unemployed individuals with mental health issues require individualized support at multiple levels in order to promote their mental health, and obtain and retain employment. The 3for1-intervention program aims to provide such support with three components (short-term psychotherapy, job coaching based on the Individual Placement and Support approach, and peer support). This study protocol outlines how the effectiveness of this three-component intervention program in terms of re-employment, mental health, and psychosocial outcomes will be evaluated.

**Methods:**

The 3for1-intervention program will be evaluated with a non-randomized controlled trial design in a multi-center study. 500 eligible participants aged between 18 and 60 years from six job centers in Southern Germany will be allocated to a control group or an intervention group. Allocation is time-based, with the control group being recruited first, followed by recruitment of the intervention group. The control group will receive treatment as usual, whereas the intervention group will receive treatment as usual as well as access to the three intervention components over a period of 12 months. Assessment will be conducted at baseline (t0), and 12 (t1) and 18 (t3) months later. The primary outcome will be the proportion of participants who are in employment subject to social insurance contributions at t1. Differences between control and intervention group will be tested with logistic regression analysis, controlling for relevant covariates. Analyses of secondary outcomes will relate to group differences regarding re-employment, health and well-being, social integration, help-seeking, and self-stigma at t1 and t2, applying logistic regression analysis or analysis of covariance. Additionally, usage of health services will be measured to evaluate the intervention program’s cost effectiveness.

**Discussion:**

The 3for1-intervention aims to improve employability and mental health outcomes of a vulnerable population with high need for assistance. Improvements for this population would benefit the German welfare state as well. This study could provide valuable insights into the feasibility, implementation, and sustainability of this individualized, multi-level support program within German job centers.

**Trial registration:**

This trial is registered with the German Clinical Trials Register: DRKS00029002 (registered on 11 May 2022).

**Supplementary Information:**

The online version contains supplementary material available at 10.1186/s12889-024-20323-0.

## Background

Unemployment and mental health are inextricably linked in a reciprocal relationship: Being unemployed is associated with poor mental health [1–3], as well as with a higher prevalence of poor physical health and sick days [1, 4, 5], a higher rate of all-cause mortality in general [4, 6] and suicide mortality in particular [7–9]. Individuals with mental health issues, in turn, are more likely to be unemployed [2, 3, 10] and struggle to obtain and retain employment in the primary labor market [11], with increasingly poor mental health as a result. Accordingly, unemployed individuals with mental health issues can find themselves in a self-perpetuating detrimental spiral.

Employment provides many benefits to individuals, such as financial security, a purpose and role in society, social interactions, status, opportunities for personal growth, and temporal structure [12, 13]. Consequently, (re-)employment has been associated with higher self-esteem and better mental health [11, 14, 15]. Accordingly, many unemployed individuals show a high level of commitment to employment and are motivated to obtain employment [16, 17], which applies to unemployed individuals with mental health issues just the same [18–20]. However, unemployed individuals with mental health issues face multiple impediments to obtaining and retaining employment: They frequently display low levels of self-esteem and various fears about returning to work [2, 17, 19], experience stigmatization due to their unemployment as well as due to their mental health issues [19, 21, 22], and are likely to perceive low self-efficacy to obtain employment [22]. Additionally, unemployed individuals with mental health issues commonly face barriers to seeking help from mental health professionals as well, such as low mental health literacy, stigmatization, or unclear options of and pathways to appropriate treatment [23]. Consequently, they cannot benefitting from available therapies in terms of their mental health [24] that, in turn, could facilitate re-employment [25, 26].

Employment is not only beneficial for individuals’ mental health, but also highly relevant to social welfare states: In Germany, for instance, costs for unemployment amounted to €60.6 billion in 2022, which includes approximately €33.5 billion for funds spent on unemployment insurance and benefits as well as about €27.1 billion for loss of revenue due to lost social insurance contributions and lost income tax revenue [27]. Based on Germany’s Second Book of the Social Code, employment services for unemployed individuals on basic income support (i.e., *Bürgergeld*) are provided by so-called job centers. There, employment advisors are tasked with helping unemployed individuals finding re-employment and reducing barriers to employment. It is estimated that over a third of recipients of basic income support in Germany have a diagnosed mental illness, with an even higher estimated number of cases of individuals who would qualify for a diagnosis of mental illness, but lack formal diagnosis, as well as individuals whose mental health issues do not (yet) amount to a formal diagnosis, but already impede functioning [28]. For these individuals, employment advisors have various ways of supporting re-employment and health such as individual coaching, psychosocial consultation, vocational rehabilitation programs, or financial incentives for employers to enable workplace inclusion. However, it appears that the integration rate of individuals with mental health issues in the primary labor market, that is, in competitive employment, remains low [29]. On the other hand, the proportion of individuals with psychological disabilities who work in sheltered workshops steadily rising [30, 31]. Especially after having worked in sheltered workshops, (re)integration rates into the primary labor market are low [32].

One possible reason for the limited integration of individuals with mental health issues in the primary labor market could be a certain level of apprehension on the part of employers. They frequently exhibit bias against job applicants with mental health issues, perceiving them as less attractive candidates and being less likely to hire them [21, 33, 34]. This may be due to the stigma attached to individuals with mental health issues in general, as well as their presumed reliability and needs in a work-related context [35]. Regarding the latter, when experiencing episodes of more severe mental health issues, work performance can be reduced [36–38] and short-term or more long-term sick leave might be necessary [5, 38]. Furthermore, some of these individuals might have certain restrictions in terms of work tasks they can fulfill as well as how, where, and for how long they can work, thus require certain accommodations at work [19, 39, 40]. However, providing reasonable accommodations for this group of individuals has been associated with low costs, received very positively by employees, and reported to be economically beneficial for employers [39, 41]. Consequently, given the relatively high prevalence of mental health issues in the general population [42–44], which makes the employment of individuals with mental health issues no isolated cases but rather likely, and the shortage of skilled workers in Germany, employers should be willing to and will likely benefit in the long-term from employing these individuals and provide reasonable accommodations to support them in contributing to the organization, the economy, and society.

Another possible reason for the limited integration of individuals with mental health issues in the primary labor market is a certain disjointedness of social services in the German social security system: While the Federal Employment Agency and the associated job centers have a mandate to promote employability and reintegration into the labor market, the health care system is responsible for restoring and maintaining physical and mental health, and if individuals are recognized as unfit for work, they are administered by the German Pension Insurance. However, many cases have complex, overlapping requirements and need support from multiple services. Collaboration between these services can be impeded as each service is regulated by different parts of the German Social Code. This results in a “jungle” of services and bureaucracy that unemployed individuals with mental health issues as well as employment advisors in the job centers can struggle to navigate, delaying suitable support [19, 28]. In response to the low rates of inclusion into the primary labor market of individuals with complex health-related barriers, the German Federal Ministry of Labor and Social Affairs has initiated a federal funding scheme, called *rehapro*, supporting the implementation of innovative proposals for better vocational inclusion and rehabilitation of these individuals at job centers and pension insurance institutions. An important feature of this funding scheme is that the funded projects have to be evaluated scientifically.

Various intervention programs that aim to facilitate re-employment and improve mental health of unemployed individuals have been developed and evaluated, with some of these specifically focusing on individuals with mental health issues or severe mental illness. Based on systematic reviews and meta-analyses, there is consistent evidence that intervention programs which focus on mental health promotion do improve participants’ mental health [45–48]. Furthermore, some of these intervention programs have been found to have beneficial effects on participants’ re-employment, but effects were small and less consistent [46, 49]. Vocational intervention programs which focus on job placement and job skills, on the other hand, have been found to increase participants’ probability of obtaining and retaining employment, but, again, effects were small and not consistent across studies [50, 51]. More consistent and positive evidence regarding employment has been found for vocational intervention programs which specifically address employment for individuals with severe mental illness through supported employment [52–54]. Furthermore, some of these vocational intervention programs have found small, but beneficial effects for participants’ mental health [45–47]. The most beneficial and consistent effects appear to result from intervention programs which combine elements that address re-employment and mental health [45, 49].

While there have been commendable intervention programs aimed at supporting re-employment and mental health for unemployed individuals, effects on obtaining and retaining employment for those with mental health issues were low and inconsistent. This may partially be attributed to factors such as intervention program’s setting, duration, or training elements used. Firstly, many of the intervention programs have been conducted in group settings, which can provide social interaction and support, but can lack individualized support. Secondly, many intervention programs have consisted of a set of training sessions over a short amount of time, rather than long-term support. Finally, most intervention programs have focused predominantly on mental health *or* re-employment and, accordingly, employing mostly training elements that address their respective focus [46]. Consequently, participants in these programs have not benefitted from combining multiple training elements.

Given that unemployment and mental health are inextricably linked, we argue that a multi-level approach over a longer time period is necessary to provide individuals with mental health issues with more individualized support in obtaining and retaining employment as well as in their mental health recovery. Consequently, and in response to the funding call of the German Federal Ministry of Labor and Social Affairs, the research project *3for1 – Drei Wege*,* ein Ziel (Three ways*,* one goal)* was proposed.

## Objectives

The 3for1-project aims to evaluate the effectiveness of a three-component intervention program, which is conducted in collaboration with six job centers in Southern Germany. The target population consists of job center clients who receive basic income support based on Germany’s Second Book of the Social Code and who experience at least moderate psychological distress. The overall goal of the intervention program is to support the target population in obtaining and retaining competitive employment in the primary labor market subject to social insurance contributions as well as to support their mental health recovery. The three components, that is, the three ways, contribute to this goal by supporting individuals on multiple levels that focus on different areas of life. Although the components have different foci, they overlap to certain extents and work jointly toward the project’s overall goal. The first component is short-term psychotherapy which is offered on a low-threshold basis and encompasses up to ten individual sessions with a psychotherapist or psychologist with clinical training. The first component focuses predominantly on mental health recovery or placement in longer-term psychiatric or psychotherapeutic treatment. The second component is job coaching based on the Individual Placement and Support (IPS) approach which supports individuals with mental health issues in obtaining and retaining competitive employment with a good personal fit [55]; the main focus here is on reintegration into the primary labor market. The final component is peer support by peer navigators. Peer navigators are individuals with lived experience of mental health issues and life crises [56]. They focus on recovery as a process and empowering individuals in their daily life to help themselves and to take control over their situation [57]. The different components can be combined in line with the clients’ needs and wishes.

We will test the hypothesis that intervention participation as a whole improves the probability of being in employment subject to social insurance contributions 12 months after study entry in comparison to a group of the same population that receives treatment as usual (TAU) within the German social security system. Furthermore, we will test the effectiveness of intervention participation in terms of secondary outcomes that relate to (re-)employment, health and well-being, social integration, help-seeking intentions and behaviors, self-stigma, and utilization of health services within the health care system. Overall, we will test whether intervention participation is associated with more favorable outcomes than non-participation. More specifically, the following nine hypotheses are proposed:

- *Hypothesis 1:* Twelve months after study entry (t1), participants in the intervention group will be significantly more likely to be in employment subject to social insurance contributions than participants in the control group.
- *Hypothesis 2: *Twelve months after study entry (t1), participants in the intervention group will have been significantly more days in employment subject to social insurance contributions than participants in the control group (H2a). For those that will have started such employment within 12 months after study entry (t1), the number of days until starting the first employment contract subject to social insurance contributions after study entry is significantly lower in the intervention group than in the control group (H2b).
- *Hypothesis 3:* Eighteen months after study entry (t2), participants in the intervention group will be significantly more likely to be in employment subject to social insurance contributions than participants in the control group.
- *Hypothesis 4: *Twenty-four months after study entry, participants in the intervention group will be significantly less likely to having been transferred to long-term basic income support than participants in the control group (long-term basic income support = at least 21 months in basic income support within the preceding 24 months).
- *Hypothesis 5: *Twelve months after study entry (t1), participants in the intervention group will report significantly higher levels of job-search self-efficacy than participants in the control group.
- *Hypothesis 6: *Twelve months after study entry (t1), participants in the intervention group will report significantly more favorable outcomes on various psychosocial measures (i.e., psychological distress, health-related quality of life, general health state, somatic symptom burden, depressive symptoms, symptoms of anxiety, social integration, self-stigma) in comparison to participants in the control group.
- *Hypothesis 7: *Twelve months after study entry (t1), participants in the intervention group will report significantly higher intentions to and past acts of seeking professional help for mental health issues than participants in the control group.
- *Hypothesis 8: *Eighteen months after study entry (t2), participants in the intervention group will report significantly more favorable outcomes on the secondary outcomes mentioned in Hypotheses 5-7 than participants in the control group.
- *Hypothesis 9: *Of those job center clients that complete the screening measure for psychological distress, at least 30% will report experiencing at least moderate psychological distress indicated by a score of nine or greater on Kessler’s Psychological Distress Scale – K6 version [58].^1^

Furthermore, to evaluate the feasibility, implementation, and sustainability of the intervention components as regular service in job centers, we will conduct qualitative interviews with relevant stakeholders (e.g., job center executive board members, employment advisors, intervention providers, and intervention participants). Finally, we will evaluate the cost effectiveness of the 3for1-intervention program.

## Methods

### Design and setting

This multi-center study is designed as a non-randomized controlled trial, with participants being allocated to either an intervention group (IG) or passive control group (CG). The group allocation is time-based, that is, there will be a first recruitment period during which all interested individuals will be allocated to the CG, followed by a second recruitment period during which all participants will be allocated to the IG.

Both groups will receive TAU in the collaborating job centers, with IG participants additionally having access to the 3for1-intervention, including its three components: (1) short-term psychotherapy, (2) IPS-based job coaching, and (3) peer support. The study will be conducted on behalf of the Jobcenter Ulm (main funding recipient and study sponsor) at the Ulm University Medical Center and the University Hospital Tübingen in collaboration with six job centers in Southern Germany. This trial was registered with the German Clinical Trials Register on 11 May 2022 (DRKS00029002).

### Inclusion and exclusion criteria

Participants will be included in the study if they fulfill the following inclusion criteria: (1) currently registered client at one of the collaborating job centers; (2) aged 18 to 60 years; (3) having received full basic income support based on Germany’s Second Book of the Social Code (i.e., *Arbeitslosengeld II* until December 2022 or *Bürgergeld* starting January 2023) up to a maximum of six months at time of study entry, or having received partial basic income support to top up income from employment subject to social insurance contributions for longer than six months, but having switched to full basic income support within the last six months due to recent job loss; (4) experiencing at least moderate psychological distress indicated by a score of nine or greater on *Kessler’s Psychological Distress Scale* – K6 version (range: 0–24) [58];^2^ (5) wish to obtain employment subject to social insurance contributions (i.e., competitive employment in the primary labor market); (6) willingness to participate in all parts of the study, including participation in the data collection and intervention participation (if offered as part of the IG); and (7) written informed consent to participate.

Participants will be excluded from the study based on (1) not fulfilling the inclusion criteria; (2) insufficient knowledge of German; and (3) currently being in employment subject to social insurance contributions or being self-employed for at least 15 working hours per week.

### Participant recruitment and timeline

Potential participants will be recruited via the employment advisors in the collaborating job centers. They will check their clients’ basic eligibility to participate in the study (i.e., inclusion criteria 1 to 3) and introduce the study’s generic premise and purpose to eligible clients during one of their first appointments in the job center. If eligible job center clients are interested and willing, they will be given the K6 to complete as a screening for their level of psychological distress. Subsequently, the employment advisors score their responses and, if they score nine or greater (i.e., inclusion criterion 4), they will be referred to the research staff located at the job center. If job center clients do not score nine or greater on the K6 and/or do not want to participate in the study, they will be asked permission to forward contact details to research staff to be contacted again in two to three months’ time to re-assess interest and/or eligibility to participate in the study (if inclusion criteria 1 to 3 are still fulfilled). Employment advisors will receive training for introducing the study to potential participants, scoring the K6 and referring participants with a K6-score of nine or greater to research staff.

Potential participants who fulfill inclusion criteria 1 to 4 will have an appointment with research staff who will inform them verbally and in writing about the study’s premise, objectives and timeline and what study participation would entail. Furthermore, they will receive information regarding the voluntary nature of their participation, data protection regulations as well as compensation for their time and travel costs. They will be given sufficient time to consider their participation and opportunity to ask questions. Subsequently, if participants remain interested, research staff will clarify whether potential participants wish to obtain employment and are willing to participate in all parts of the study (i.e., inclusion criteria 5 and 6), and, finally, obtain written consent of participants (inclusion criterion 7).

Upon study entry, research staff will record detailed contact information from participants as well as their banking details for compensation, including written consent to use these details for said purpose. Subsequently, research staff will conduct an entry interview with participants and ask them to complete a questionnaire (i.e., baseline assessment, t0). After completing this baseline assessment, participants will be contacted for a short telephone interview after six months to assess health service utilization in the preceding six months, as well as after 12 months (i.e., t1) and 18 months (i.e., t2) for more in-depth assessment similar to the baseline assessment, including conducting an interview and completing a questionnaire. Additionally, research staff will ask participants’ written consent to request certain data from their job center records, specifically their status of receiving basic income support benefits (yes vs. no) and the duration of receiving full basic income support at 12, 18 and 24 months after study entry. Whether written consent for this additional data request is given or not will not affect study participation.

**Fig. 1:**
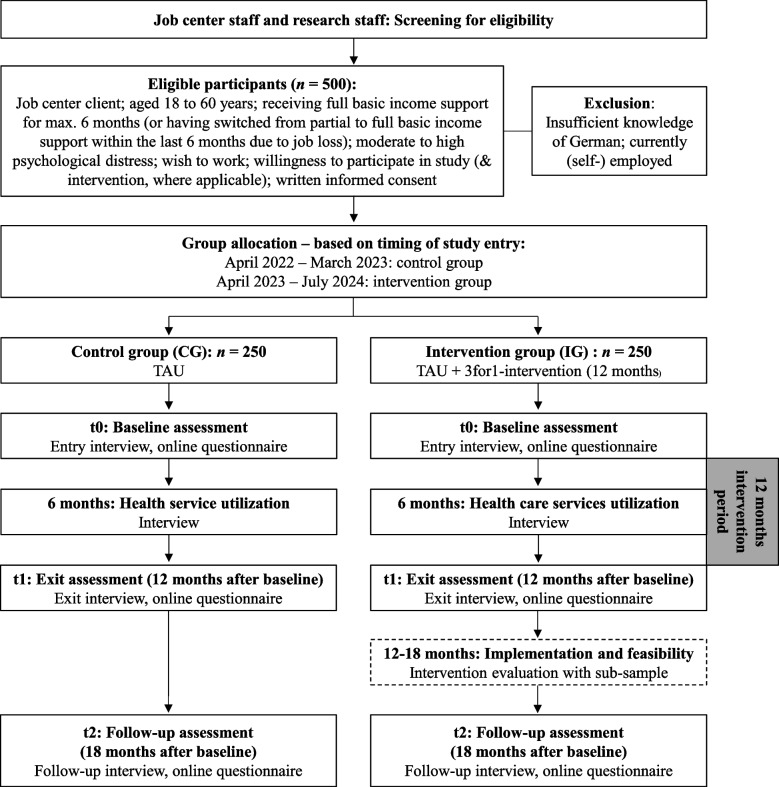
Study flow for participants in the 3for1-project

Figure 1 shows a flow chart for participants’ study experience.

At the time of the initial manuscript submission, the recruitment phase for this study was ongoing, but not yet completed. Prior to recruiting the first participant, this study, its objectives and hypotheses were pre-registered with the German Clinical Trials Register: DRKS00029002 (registered on 11 May 2022).

### Sample size

We aim to recruit 500 participants, 250 participants per group. Sample size calculation is based on the primary outcome measure at t1 (i.e., employment status 12 months after study entry). Based on a previous longitudinal observational study with unemployed individuals with mental health issues [61], we assume a re-employment rate of 15% with TAU. We aim to achieve a re-employment rate of 30% in the IG. We conducted an a-priori power calculation for this expected intervention effect [62]: Applying a two-sided significance level of α = 0.05 and a statistical power of 0.90, we estimated that *n* = 161 participants in each group are required to show this effect. Based on previous research projects in this subject area, we anticipate a dropout rate of 35% between t0 and t1. To test the intervention effect for the primary outcome, we accordingly require an initial sample size of at least *n* = 248 participants per group at t0.

### Intervention

Participants of both groups will receive TAU within the German social security system; there are no restrictions in terms of concomitant care. This could mean, for instance, that individuals participate in training programs provided by the job center (e.g., application training) or draw on health services such as psychotherapy or medication.

In addition to TAU, the IG will be offered to participate in the 3for1-intervention program and its three components over 12 months after study entry. For intervention utilization, certain time or session limits will be applied in the different components (see below for further details). Participants will be able to choose which of the components they utilize and when: They can choose one, two or three components, concurrently or consecutively. They can make the decision based on their individual needs in order to support self-determination and facilitate intervention motivation and adherence. They will be advised by the local intervention team in their decision. This means that the intervention provider of one component might suggest a suitable other component to utilize concurrently or consecutively based on their experience with the individual participant. The final decision will always lie with the participant. Participation in the intervention overall and the individual components will be voluntary and can be terminated or paused at all times during the 12-months intervention phase. Terminating or pausing participation in one component will not have negative consequences for the participation in the other components.

#### Intervention components

##### Short-term psychotherapy

Participants can receive up to 10 sessions of short-term psychotherapy with a psychotherapist or psychologist with clinical training. Each session will last up to 50 min, which is equivalent to a regular psychotherapy session provided in the German outpatient health care system. In addition to a comprehensive anamnesis and clarifying the participants’ goals of the short-term psychotherapy, the initial sessions will focus on promoting motivation for change. The content of subsequent sessions may vary depending on the formulated goals and can include psychoeducation, developing a bio-psycho-social model of mental illness, resource work, developing a daily or weekly routine, or promoting problem-solving skills, self-efficacy, or social and emotional competencies. Promoting and maintaining motivation to change will be a topic throughout all sessions. Additionally, the indication for further treatment will be assessed. Accordingly, participants may receive a recommendation for further treatment and assistance in finding an outpatient or inpatient therapy place. In the last two sessions, the psychotherapy sessions are reviewed together and the need for booster sessions is assessed. Booster sessions could be indicated for the following goals: relapse prevention, maintenance of treatment gain, refresh learned skills, (re-)assessment of indication for further treatment, or support until continuing treatment starts. Up to three booster sessions can be offered on a case-by-case basis; indication will be discussed in supervision sessions. In general. they will be offered no sooner than two months after the last regular session.

The 3for1-component of short-term psychotherapy will apply an integrative psychotherapeutic approach that combines cognitive-behavioral, systemic, and psychodynamic methods and techniques. According to the competence structure for counseling for depression developed as part of the *Improving Access to Psychological Therapies* program [63], the focus is on therapeutic competencies rather than the selection of specific psychotherapy directions. The short-term psychotherapy component is designed based on the well-established psychotherapeutic consultation in the workplace. These workplace outpatient clinics have been tested as a work-related short-term psychotherapy intervention program and found to be beneficial in terms of symptom reduction [64]. In addition to reducing symptoms specific to certain mental illnesses, the short-term psychotherapy also includes psychotherapeutic methods focused on facilitating re-employment. Findings of previous intervention studies using psychotherapeutic methods in the workplace context indicate the effectiveness and efficiency of such intervention programs in terms of shorter duration until return to work [65, 66]. An important element of the short-term psychotherapy within the 3for1-project is the provision of low-threshold service where individuals can seek counseling without long waiting times and without formal record of utilization with the health insurance.

Each local intervention team will include a 50% full-time equivalent (FTE) psychotherapist or psychologist with clinical training. They will receive intervention-specific training, weekly group supervision, and individual supervision if required. Supervision will be provided by certified psychological psychotherapists and psychiatrists who are members of the research project team.

##### Job coaching

Participants can receive job coaching based on the IPS approach [55, 67]. The IPS approach is a vocational rehabilitation program for individuals with severe mental health issues. The approach implements the principle of *first place*,* then train*, meaning that the job coaching aims to place individuals relatively rapidly in competitive employment in the primary labor market and then trains them at the workplace. In traditional vocational rehabilitation programs, individuals are trained for a considerable amount of time to prepare them for re-entering the workplace. After successful job placement, no further support is usually provided. Based on meta-analyses and reviews, the IPS approach is superior to traditional vocational rehabilitation programs for individuals with severe mental health issues in terms of obtaining and retaining competitive employment [52–54]. IPS job coaching focuses on individual needs and preferences, aiming to find a job and workplace that fits the individual. IPS job coaching involves all aspects of obtaining and retaining employment such as assessment of needs, capabilities, and competencies, individual coaching to obtain employment (e.g., sifting through job offers, creating or revising application documents, accompanying individuals to job interviews), individual coaching to stay in employment (e.g., conducting workplace visits to discuss required adjustments, mediating in conflicts that might arise at the new workplace) as well as building a network of local employers in order to match individuals with suitable employers. IPS job coaching can also include vocational re-orientation. In terms of accompanying individuals to employers, both potential and actual, individuals can always choose for themselves whether they want to disclose their mental health issues to employers; hence they can decide how they would like to explain the job coach’s presence. Discussing the advantages and disadvantages of disclosing mental health issues with individuals is an important part of IPS job coaching as individuals might find employment more easily if not disclosing mental health issues, but then in the long run missing out on reasonable accommodations by an unaware employer [61]. If individuals lose or quit their employment, IPS job coaches discuss the underlying reasons in order to revise their assessment to find a better fitting job in the future. In the 3for1-intervention, participants can receive up to 25 h of job coaching to obtain employment [68]; after obtaining employment, job coaching is unlimited within the 12-months intervention period.

Each local intervention team will include an 80% FTE job coach. All job coaches will work manual-based [55]. They will receive an online training course (https://ipsworks.org/index.php/training-courses/) and a one-day, in-person training course from a German expert in IPS job coaching as well as participate in workplace shadowing in an established IPS unit. Throughout the intervention, they will receive weekly group supervision and individual supervision if needed. Supervision is provided by the same project-internal individuals that provide supervision to the other components. Additionally, job coaches will receive a monthly supervision session by the aforementioned IPS expert external to the team.

##### Peer support

Peer navigators are individuals with lived experience of mental illness and life crises [56]; many have experienced episodes of unemployment, reduced earning capacity or disability pensions. Peer navigators are experts through experience rather than having professional training in health care or social care. An important component of peer support is the disclosure of lived experience with mental health issues in order to create shared lived experience and build a rapport with individuals with mental health issues seeking help [56, 69]. They discuss participants’ needs and aims with them and how they can be fulfilled; in doing so, they focus on individual recovery as a process and empowerment [57]. Peer navigators support participants emotionally and instrumentally in navigating their daily activities, especially within the health care system, social security system, or visits to official authorities. This includes, among others, that peer navigators accompany individuals to appointments with health professionals, in the job center, or debt counseling. Other activities can include activation in daily life (e.g., going for a walk, starting a new hobby) and building a social network (e.g., by joining a self-help group). Peer navigators follow the principle of supporting individuals on equal footing, showing them how they can take control over their lives and help themselves. Peer support is unlimited within the 12-months intervention period.

There will be four peer navigators per local intervention team; each peer navigator will be employed on a marginal part-time employment, which is equivalent to around seven work hours per week. All peer navigators work manual-based (adapted from: [70]) and receive a 1.5-day, in-person training course. Parallel to the other components, they also receive weekly group supervision and individual supervision if required. Supervision is provided by the same individuals as the other components.

##### Integration of components

Each local intervention team consists of a 50% FTE psychologist/psychotherapist, an 80% FTE job coach, and four peer navigators on marginal part-time employment as intervention providers, as well as a 50% FTE researcher who conducts the study onboarding and data collections, and coordinates the local intervention providers. The researcher and intervention providers work as a local team with weekly team meetings where cases are discussed. All team members have equal rights and are encouraged to contribute their own perspective to the cases. Involving other component providers in the cases, where suitable, is encouraged to provide effective individualized support to participants.

#### Adherence and auditing

Participants can determine which components they use, in what combination and for how long over the 12-months intervention phase. Intervention providers will offer participants regular appointments (i.e., every one to two weeks) with low-threshold scheduling to enable regularity and structure. Although face-to-face appointments are preferred, also in terms if relationship building, intervention providers will offer online appointments as well where necessary. To facilitate face-to-face appointments, reimbursement of travel costs is offered.

Participating in a component can be paused or terminated within the time frame of the 12-months intervention phase. If participants state they would like to pause or terminate participation in one component, intervention providers will stress that the access to that component remains open within the 12-months period and encourage participants to contact intervention providers if their needs change and they would like to resume the component. Additionally, intervention providers will continuously encourage participation in the other two components where considered beneficial. If participants do not use any component for a certain time, research staff will regularly contact them to offer support via the intervention, potentially re-iterating the intervention components.

The intervention providers’ conduct and adherence to intervention manuals will be discussed in regular supervision meetings. Furthermore, every six months, there will be a fidelity assessment for each intervention component to review and, if necessary, further develop high fidelity in each component. The fidelity assessment will be conducted by an internal and an external reviewer based on predefined fidelity scales. Subsequent fidelity reports and emerging barriers will be discussed with the principal investigators and measures taken to improve intervention fidelity further if required.

### Assessments

Upon study entry, baseline assessment includes data collection on a variety of sociodemographic variables via a structured entry interview and an online questionnaire. These variables include age, gender, country of birth, citizenship(s), ethnicity, marital status, number of children, household income and debts, educational background, sick leave days, and history of homelessness. Additionally, participants will be asked questions in relation to their current unemployment status, employment history, and attempts and barriers to re-employment within the last 12 months. In relation to mental health, participants are asked questions in terms of mental health history (e.g., diagnoses, inpatient stays in a psychiatric or psychosomatic clinic), currently experienced symptoms and used treatment for mental health issues. The entry questionnaire also includes a screening for drug and alcohol abuse with the 4-item *CAGE-AID* instrument [71, 72].

After baseline assessment, further in-depth assessment takes place 12 months (t1; exit assessment) and 18 months (t2; follow-up assessment) after study entry, including structured interviews and online questionnaires. Additionally, every six months, participants are interviewed on their health service utilization as part of the cost effectiveness evaluation.

#### Primary outcome

The primary outcome is the proportion of participants who are in competitive employment in the primary labor market subject to social insurance contributions 12 months after study entry (yes vs. no). Competitive employment in the primary labor market refers to employment for which anyone can apply and which is not protected or accessible for individuals with disability status only. It will be assessed via self-report as part of the exit interview.

#### Secondary outcomes

Secondary outcomes encompass variables in relation to (re-)employment, health and well-being, social integration, help-seeking, and self-stigma. These variables will be assessed at t0, t1 and t2 by self-report, predominantly via online questionnaires, but also as part of structured interviews.

We will collect data on various variables related to (re-)employment. We will apply a one-item measure to ask participants about their *motivation to perform paid work* in the primary labor market [20], ranging from 1 (= *low*) to 3 (= *stron*g), with 4 (= *not applicable*,* as currently employed*) specifically for assessment at t1 and t2. Additionally, we will measure how confident participants feel in finding a job, namely job-search self-efficacy (JSSE). Variables regarding current employment status will be measured as self-report at t1 and t2 only (as the inclusion criteria at t0 preclude current employment). More specifically, participants will be asked in the exit and follow-up interview about employment they have started since entering the study or since the exit interview respectively. These questions encompass start and end date of potential employment, type of employment (e.g., part- or full-time employment, self-employment), working hours per week and average monthly net income. Finally, participants will also be asked about measures taken to obtain re-employment since the last assessment.

Furthermore, we will assess the following variables related to participants’ health and well-being in questionnaire format with established measurement scales: psychological distress, health-related quality of life, general health state, general well-being, somatic symptom burden, depressive symptoms, and symptoms of anxiety. Another set of measurement scales will assess participants’ social inclusion and social outcomes in relation to employment, housing situation, and social contact. Additionally, participants’ willingness to seek professional help for mental health issues and actual help-seeking behaviors in the last six months will be assessed. Finally, we will assess to which extent participants apply stigma toward mental health issues to themselves. As we will include participants who do not necessarily have a formally diagnosed mental illness, we change the introductory clause from the applied scale by Corrigan et al. [73] from “Because I have a mental illness, I …” to “Because I have mental health issues, I …”, for instance, “… will not recover or get better”.

Table 1 provides an overview of the employed measurement scales.

**Table 1:** Overview of employed measurement scales for outcome variables

Variable	Scale	Scale source(s)	No. of items	Response range	Scale scores and interpretation	Reliability score(s)
Job-search self-efficacy	Job-Search Self-Efficacy Scale (JSSE)	[74]	6	1 = *not at all confident* to 5 = *great deal confident*	1–5 (mean score); higher score = higher level of job-search self-efficacy	α > 0.85 [22, 74, 75]
Psychological distress	Kessler’s Psychological Distress Scale (K6)	[58, 76]	6	0 = *none of the time* to 4 = *all of the time*	0–24 (sum score); higher score = higher level of psychological distress	α = 0.89-0.92 [58]
Health-related quality of life	EQ-5D-3 L	[77]	5	1 = *no problems* to 3 = *extreme problems*	The five responses are combined to present one of 243 possible health state profiles and evaluated based on country-specific value sets [78]	n/a
General health state	EQ-VAS	[77]	1	0 = *worst health* to 100 = *best health*	0-100; higher score = higher level of general health	n/a
General well-being	World Health Organization Well-Being Index (WHO-5)	[79, 80]	5	0 = *at no time* to 5 = *all the time*	0–25 (sum score); higher score = higher level of general well-being	α = 0.92 [80]
Somatic symptom burden	Somatic Symptom Scale (SSS-8)	[81, 82]	8	0 = *not at all* to 4 = *very much*	0–32 (sum score); higher score = more somatic symptoms	α = 0.81 [82]
Depressive symptoms	Patient Health Questionnaire (PHQ-9)	[83–85]	9	1 = *not at all* to 4 = *almost every day*	0–27 (sum score); higher score = more depressive symptoms	α = 0.88 [86]
Symptoms of anxiety	Generalized Anxiety Disorder Screener (GAD-7)	[87, 88]	7	0 = *not at all* to 3 = *nearly every day*	0–21 (sum score); higher score = more anxiety symptoms	α = 0.89 [88]
Social inclusion	Experiences of Social Inclusion Scale (ESIS)	[89]	10	1 = *strongly disagree* to 5 = *strongly agree*	10–50 (mean score); higher score = higher level of social inclusion	α = 0.89 [89]
Social outcomes	Social Outcomes Index (SIX)	[90]	4	Employment and living situation: 0 = [least preferable] to 2 = [most preferable Partnership/family and friends: 0 = [least preferable] to 1 = [most preferable]	0–6 (sum score); higher score = more preferable social outcomes	n/a
Help-seeking	General Help-Seeking Questionnaire (GHSQ) – adapted	[91, 92]	3	1 = *extremely unlikely* to 7 = *extremely likely*	3–21 (sum score); higher score = stronger intention to seek help from mental health professionals, general practitioners, or no one (reversed)	n/a (adapted)
Help-seeking	Actual Help-Seeking Questionnaire (AHSQ)	[92, 93]	2	0 = *no* 1 = *yes*	Acts of help seeking from mental health professionals evaluated individually	n/a
Self-stigma	Self-Stigma of Mental Illness Scale – Short Form (SSMIS-SF) – Subscale Apply	[73]	5	1 = *strongly disagree* to 9 = *strongly agree*	5–45 (sum score); higher score = higher degree of applying stigma to oneself	α = 0.74 [73]

As part of the t1 online questionnaire, we will ask IG participants to evaluate their intervention experience. We will first ask them to rate their overall satisfaction with the intervention on a visual analogue scale, ranging from 0 (= *lowest satisfaction*) to 100 (= *highest satisfaction*). Secondly, IG participants will be asked to rate their satisfaction with the intervention components in which they have participated in the intervention phase. For this rating, we adapt a questionnaire for client satisfaction with hospital stays (ZUF-8) [94] to fit our intervention components. Satisfaction with each intervention component will be evaluated with eight items; each item offering four response options. Thirdly, we will apply seven self-created items for each component to evaluate to which extent the components are considered helpful by participants for different aspects of their life (e.g., to change their situation, to find employment, to improve mental health). Each item will be rated in a 5-point Likert scale from 1 (= *strongly disagree*) to 5 (= *strongly agree*).

Additionally, we will assess health service utilization every six months with the *Client Socio-Demographic and Service Receipt Inventory* (CSSRI) [95] (German version: [96]) in order to evaluate the cost effectiveness of the 3for1-intervention program. The CSSRI will be completed via structured interviews, either as part of the interviews at t0, t1 and t2 or an additional interview six months after study entry.

In addition to the questionnaires and structured interviews, we will inquire data from job center records whether participants receive full basic income support benefits and for how many months they have received full benefits since study entry at 12, 18 and 24 months after study entry.

Finally, we will record the intensity of IG participants’ intervention utilization in our database: Intervention providers will record contact times (in minutes) with participants after every intervention session, including an overview of discussed topics. Based on these recorded contact times, we will calculate overall intervention utilization intensity as well as intervention component utilization intensity to explore whether more intensive utilization of the intervention and its components is associated with its effectiveness.

An overview of enrollment, intervention, and assessments across the different measurement points is given in Table 2.

**Table 2:** Overview of enrollment, intervention, and assessments in the 3for1-project across measurement points

	**STUDY PERIOD**					
	**Enrollment**	**Baseline**		**Exit**	**Follow-up**	
TIMEPOINT^*^	***t0***	***t0***	***6 months***	***t1***	***t2***	***24 months***
**ENROLLMENT:**						
**Eligibility screen (incl. psychological distress)**	X					
**Project information**	X					
**Informed consent **	X					
Allocation^**^	X					
**INTERVENTIONS:**						
**3for1 components**		X	X	X		
**Control group**		X	X	X		
**ASSESSMENTS:**						
**Questionnaires:**						
Sociodemographic variables		X		X	X	
Motivation to work		X		X	X	
Job-search self-efficacy (JSSE)		X		X	X	
Psychological distress (K6)				X	X	
Health-related quality of life (EQ-5D)		X		X	X	
General health state (EQ-VAS)		X		X	X	
General well-being (WHO-5)		X		X	X	
Somatic symptom burden (SSS-8)		X		X	X	
Depressive symptoms (PHQ-9)		X		X	X	
Symptoms of anxiety (GAD-7)		X		X	X	
Mental health diagnoses		X		X	X	
Mental health treatment		X		X	X	
Social outcomes (SIX)		X		X	X	
Social inclusion (ESIS)		X		X	X	
Help-seeking (GHSQ; AHSQ)		X		X	X	
Self-stigma (SSMIS-SF)		X		X	X	
Intervention evaluation (IG only)				X		
**Structured interviews:**						
Sociodemographic variables / changes in these variables		X		X	X	
(Un-) Employment history		X				
Current/recent employment				X	X	
Attempts on re-employment		X		X	X	
Barriers to re-employment		X				
Mental health issues & treatment		X		X	X	
Health service utilization		X	X	X	X	
**Job center records:**						
Receiving benefits: yes vs. no				X	X	X
Duration of receiving benefits after study entry (in months)				X	X	X
**Database records (IG only):**						
Intervention utilization intensity (in minutes)				X		
Intervention component utilization intensity (in minutes)				X		

^*^t0: Enrollment and baseline assessment at the same timepoint, individual intervention period of 12 months for IG starting directly afterward; t1: exit interview and questionnaires 12 months after enrollment and baseline, and, for IG, directly after end of intervention period; t2: follow-up interview and questionnaires 18 months after enrollment and baseline, and, for IG, 6 months after end of intervention period

^**^Allocation based on enrollment timing in study: allocation of the first cohort to CG and the second cohort to IG

### Data collection and management

Assessments will be conducted via structured interviews and online questionnaires at all measurement points. We aim to conduct most interviews in person, but will conduct them via telephone if required and more practical. In particular, the interview six months after study entry on health service utilization will be conducted predominantly via telephone because its short duration would be disproportionate to the travel time to the research sites. Online questionnaires can be completed either on a tablet computer directly in the job center or via a link sent by e-mail. Paper-pencil-versions will be provided as well, in particular for rare technical issues, parallel assessments per research location, or participants with low technological proficiency. Research staff will assist questionnaire completion if needed.

We will employ multiple processes to ensure high quality and completeness of the collected data. Firstly, we selected established instruments for the questionnaires, many have been successfully used in a previous study in a similar population [61]. Regarding employed interview questions, they were discussed and assembled in an iterative process within our research team whose principal investigators have in-depth research experience in the topic area. Secondly, research staff will be trained to administer the questionnaires and conduct the interviews. They will be present when participants complete questionnaires and support them where needed. After measurement points, research staff will check the participants’ responses for completeness, data input mistakes, and apparent inconsistencies. If necessary, they will resolve these together with participants. Thirdly, research staff will meet regularly together with the project coordination and principal investigators to discuss the data collection and to clarify any open questions regarding special cases. This process will ensure high consistency in data collection across the different research sites. Finally, data quality checks will be performed on a regular basis by the biometrical experts within the research team to verify the completeness and plausibility of the data input in the database. Any anomalies in the database will be discussed and resolved within the research team.

### Statistical methods

For the analysis of the primary outcome, logistic regression analysis will be applied. Logistic regression analysis enables the prediction of the binary outcome (in employment: yes vs. no) based on the binary group status (CG vs. IG), while controlling for relevant covariates such as the job center that the participant is registered with, the unemployment rate of the respective districts, duration of unemployment and of receiving basic income support, as well as baseline demographic variables which are significantly different in the two groups. The secondary outcomes will be analyzed with varied analysis methods based on the characteristics of the data: For the hypotheses which propose differences in binary outcomes (i.e., Hypotheses 3–4), logistic regression analysis will be applied; for hypotheses that predict difference in continuous outcomes (i.e., Hypotheses 2, 5–8), we will apply analyses of covariance to compare the two study groups. To estimate the prevalence of increased psychological distress in job center clients that complete the screening measure, we will conduct a frequency analysis (i.e., Hypothesis 9).

Additionally, we will analyze the cost-effectiveness of the intervention from two perspectives: We will address the intervention’s cost effectiveness in terms of benefits of employment for the social welfare state. More specifically, we will balance the costs for the intervention with the reduced costs for basic income support and related social welfare benefits. Furthermore, we will evaluate the intervention’s cost-effectiveness by relating the incremental total costs of illness, including the direct costs of health service utilization and the indirect costs of productivity loss, to the incremental gain in quality adjusted life years (QALYs) [97, 98].

Analyses will be conducted using all observations with available data, applying an intention-to-treat-approach. Accordingly, participants will be treated as part of the group to which they were allocated at study enrollment. As the study spans over 1.5 years, we expect a certain degree of dropouts; we accounted for a 35% dropout rate between t0 and t1 in our power analysis. Research staff will make every reasonable effort to obtain as complete data as possible from as many participants as possible across all measurement points, especially regarding the primary outcome measure. To incentivize participation in the assessments, we will offer compensation for time and travel costs. At study enrollment, we will collect multiple ways to contact participants (i.e., postal address, phone number, mobile number, multiple e-mail addresses, contact information for significant others). After enrollment, we will maintain regular contact with participants to facilitate up-to-date contact information for the next assessment. If data cannot be collected at one measurement point after study enrollment (e.g., t1), we will attempt to collect data at the following measurement point.

In order to reduce missing data on individual items, participants will be notified of missing responses by the database and encouraged to provide complete responses. If questionnaires are answered on paper, research staff will review them immediately and ask the participant to complete any missing items. If items are still missing, we will use multiple imputation if up to 30% of item responses are missing; if a higher percentage is missing, no scale score is calculated.

#### Interim analyses

Interim analyses will be performed after the completion of the second measurement point (t1) to evaluate the intervention program’s effectiveness in terms of the primary outcome. The second measurement point is expected to be completed in June 2025. These interim analyses are particularly relevant for the annual reports that the research team provides to the project funder. The study will not be modified or terminated based on interim analyses.

### Ethical considerations

#### Protocol amendments

All major changes to the study protocol that may affect the conduct of the study, intervention design, outcomes, or participant safety will be submitted as an amendment to the respective ethics committee of the involved research institutions for approval and subsequently communicated by the project coordination to all relevant parties through regular planning meetings, direct contact, and newsletters. The registration with the German Clinical Trials Registry (DRKS00029002) will be updated according to amendments. This study protocol is the second major version (27 March 2023).

#### Adverse events

We consider the risk of adverse effects from participating in the study’s data collection to be low, apart from potentially short-term discomfort of participants while reporting and discussing their unemployment and psychological well-being.

Participation in the intervention program will be voluntary and can be paused or terminated at all times during the 12-months intervention phase. The intervention program is explicitly aimed at supporting participants’ re-employment and mental health recovery so we consider the risk of adverse effects through participation to be low. Research staff and intervention providers will continuously monitor for serious adverse events. Any serious adverse events will be discussed in regularly scheduled clinical supervision sessions and their occurrence will be documented in the session records. Furthermore, participants are provided with contact details of research staff to report adverse events. Research staff and intervention providers are provided with clear guidelines if participants were to express thoughts and plans of self-harm. As we consider the risk of adverse effects of intervention participation to be low, we will conduct regular data monitoring internally; no independent data monitoring committee will be formed.

#### Confidentiality

The collection, storage, and analysis of study data will be carried out in strict compliance with the General Data Protection Regulation of the European Union (DSGVO). Study data will be entered and stored in the project’s database secuTrial^®^ (interActive Systems Berlin). This database is password-protected both for participants and researchers and provides an audit trail. It is certified according to the guidelines for Good Clinical Practice of the International Council for Harmonization of Technical Requirements for Pharmaceuticals for Human Use. Each participant will be allocated a pseudonym (project-ID) by the database that allows us to connect study data from the various measurement points. The documents that connect personal data (e.g., contact details) with project-IDs will be password-protected and stored separately from the study data. Accordingly, datasets extracted from the database will be analyzed in a strictly pseudonymous form. Results will be published in anonymous and aggregated form. After project completion, data will be archived on servers of the two involved research institutions. Data which are collected on paper (e.g., interviews notes, signed consent forms, bank detail for compensation) will be stored separately form the study data in secure locations. All data related to the study will be stored securely by the involved research institutions for 10 years after study completion and subsequently deleted or destroyed. Digital data will be stored within the server infrastructure of the research institutions; physical data (e.g., signed consent forms) in locked file cabinets in areas with limited access.

Datasets that are extracted from the database will be password-protected and accessible only to the study’s biometrical experts within the research team, principal investigators, and the project coordination, as well as to research staff who express a legitimate interest in the data for further academic qualification and will have signed a data use agreement.

### Dissemination

The research team will publish all findings regarding the intervention’s effectiveness, implementation, and sustainability as permanent service in job centers via multiple pathways—independently from the funder, sponsor, or the collaborating job centers and regardless of strength and direction of the effects, including non-significant findings. The findings will be published in peer-reviewed scientific journals and presented at relevant national and international conferences. Authorship eligibility will be based on the recommendations outlined by the International Committee for Medical Journal Editors (ICMJE).

The funder will receive regular reports regarding project progress, including interim and final findings. Participants and staff of collaborating job centers will be informed about the study’s final findings through newsletters and an executive summary; this executive summary will also be published on the project’s public website (https://www.projekt-3for1.de). Additionally, findings will be disseminated as press releases to various media outlets. We will approach political decision-makers, multiplier institutions (e.g., Federal Employment Agency, chambers of industry and commerce, community psychiatric associations), and interest groups (e.g., non-profit organizations for individuals with lived experience) with the findings.

## Discussion

Unemployment incurs large costs for social welfare states and is detrimental to those affected, making it a significant public health concern. Unemployed individuals with mental health issues—partially as result of their unemployment—often desire to re-enter the labor market, but struggle to do so, experiencing multiple barriers. This is where the 3for1-project comes in, offering a multi-level support program that addresses both re-employment and mental health recovery in German job centers. By offering support on different levels, the proposed program fills an important gap in both research into unemployment and mental health, as well as in practice in job centers who are tasked to facilitate re-employment of basic income support recipients.

Our study has several strengths. One strength is that our intervention program addresses multiple areas of life that impede unemployed individuals with mental health issues from finding re-employment: obtaining and retaining employment, improving mental health, facilitating self-efficacy and self-determination, increasing social networks and activation level in daily life. The intervention program offers highly individualized support over a 12-months period to a high-risk population with complex needs for assistance. Furthermore, we will be able to compare the IG with a CG from the same population over multiple measurement points, including a 6-months follow-up assessment. Another strength is the recruitment of a substantial sample from six collaborating job centers—with some job centers being responsible for a city, others for partially rural districts. This design enhances the reliability and generalizability of the study’s findings.

Despite the innovative and comprehensive approach of our intervention program, our study has certain limitations. Firstly, the study groups are not randomized, but allocated based on time. As the 3for1-intervention program includes active cooperation with the job centers’ employment advisors, we expect that the culture and way of working in the job centers themselves could be changed by the intervention. Therefore, a parallel CG could be biased by these changes, impeding the evaluation of the intervention’s actual effectiveness. However, the non-randomization could mean that the two groups differ on demographic variables and relevant outcomes at baseline. Additionally, as the recruitment phase for both groups will cover several years, there may be various developments in society and policies that could affect the two groups differently. We will collect a variety of covariates and use analysis methods that enable us to control for these covariates, such as logistic regression or analysis of covariance, to minimize bias in the estimation of the intervention effect [99]. Another challenge will be participant retention across the 1.5-year study period. If participants drop out of the study, conclusions from the analysis might be biased by selective dropout (e.g., less motivated participants, participants in CG without offered benefit of the intervention program). As described, we will make every reasonable effort to collect data from as many participants as possible across the study period. Additionally, we will analyze the data in terms of selective dropout effects. Finally, insufficient German language skills are an exclusion criterion. Consequently, the effectiveness of the intervention program can only be evaluated for a subsample of unemployed individuals with mental health issues. Therefore, we can only draw limited conclusions about its broader effectiveness for job center clients with mental health issues in general.

Despite certain challenges of our study design, the 3for1-intervention program has great potential to be a valuable addition to the German job center infrastructure, offering support for a vulnerable group with a high need for assistance. Particularly, with the K6, job centers would be equipped with a reliable, quick and easy-to-use screening tool to identify this group. As part of the project, we will have implemented relevant administrative infrastructure within the job centers and deduced multiple best practices for implementation. These aspects will be invaluable in facilitating the sustainability of the intervention program within the job centers as a regular support offer for clients with mental health issues.

## Supplementary Information

Supplementary Material 1.

## Data Availability

No datasets were generated or analysed during the current study.

## Abbreviations

AHSQ: Actual Help-Seeking Questionnaire
CG: Control group
CSSRI: Client Socio-Demographic and Service Receipt Inventory
DSGVO: Datenschutz-Grundverordnung (General Data Protection Regulation)
EQ-5D-3L: EuroQol Group’s instrument to measure health-related quality of life
EQ-VAS: EuroQol Group’s visual analogue scale for self-rated health state
ESIS: Experiences of Social Inclusion Scale
FTE: Full-time equivalent
GAD-7: Generalized Anxiety Disorder Screener
GHSQ: General Help-Seeking Questionnaire
ICMJE: International Committee for Medical Journal Editors
IG: Intervention group
IPS: Individual Placement and Support
JSSE: Job-search self-efficacy
K6: Kessler’s Psychological Distress Scale – 6-item version
PHQ-9: Patient Health Questionnaire
QALYs: Quality-adjusted life years
SIX: Social Outcomes Index
SSMIS-SF: Self-Stigma of Mental Illness Scale – Short Form
SSS-8: Somatic Symptom Scale
TAU: Treatment as usual
WHO-5: World Health Organization Well-Being Index

## Footnotes

1 This hypothesis does not refer to the evaluation of the intervention’s effectiveness, but to examine the prevalence of psychological distress in job center clients that register for basic income support.

2 We implement a score of nine or greater on the K6 based on Cornelius et al. [59] who calculated this optimal cut-off score for screening for probable mental illness in a population of disability claimants, arguing that established cut-off scores are based on general population [60] and are not directly applicable to vulnerable populations in which mental health issues are commonly under-recognized.
